# Design of a Hybrid Indoor Location System Based on Multi-Sensor Fusion for Robot Navigation

**DOI:** 10.3390/s18103581

**Published:** 2018-10-22

**Authors:** Yongliang Shi, Weimin Zhang, Zhuo Yao, Mingzhu Li, Zhenshuo Liang, Zhongzhong Cao, Hua Zhang, Qiang Huang

**Affiliations:** 1 School of Mechatronics, Beijing Institute of Technology, Beijing 100080, China; ylshi@bit.edu.cn (Y.S.); 2220170106@bit.edu.cn (Z.Y.); limingzhu@bit.edu.cn (M.L.); 2220170089@bit.edu.cn (Z.L.); czz1410407667@163.com (Z.C.); qhuang@bit.edu.cn (Q.H.); 2 Key Laboratory of Biomimetic Robots and Systems, Beijing Institute of Technology, Ministry of Education, Beijing 100080, China; 3 Beijing Advanced Innovation Center for Intelligent Robots and Systems, Beijing 100080, China; zzhanghua@163.com

**Keywords:** indoor localization, multi-sensor fusion, rough localization, KNNBP, precise localization, HPFL

## Abstract

In the case of a single scene feature, the positioning of an indoor service robot takes a long time, and localization errors are likely to occur. A new method for a hybrid indoor localization system according to multi-sensor fusion is proposed to solve these problems. The localization process is divided in two stages: rough positioning and precise positioning. By virtue of the K nearest neighbors based on possibility (KNNBP) algorithm first created in the present study, the rough position of a robot is determined according to the received signal strength indicator (RSSI) of Wi-Fi. Then, the hybrid particle filter localization (HPFL) algorithm improved on the basis of adaptive Monte Carlo localization (AMCL) is adopted to get the precise localization, which integrates various information, including the rough position and information from Lidar, a compass, an occupancy grid map, and encoders. The experiments indicated that the positioning error was 0.05 m; the success rate of localization was 96% with even 3000 particles, and the global positioning time was 1.9 s. However, under the same conditions, the success rate of AMCL was approximately 40%, the required time was approximately 25.6 s, and the positioning accuracy was the same. This indicates that the hybrid indoor location system is efficient and accurate.

## 1. Introduction

It is required that an indoor service robot always accurately estimates its own position in order to complete its task efficiently. Generally, an indoor wheel-based mobile robot estimates its own position by integrating wheel encoders and Lidar [1]. However, when in an environment with a low degree of distinction (e.g., a long corridor or hall), robot positioning is time-consuming and has low accuracy.

To solve these problems, numerous approaches that use inertial sensors, ultrasonic, radio-frequency (RF) ID tags, Wi-Fi, Lidar, vision sensors, and so on have been proposed [2,3,4,5,6,7,8]. Grid localization and Monte Carlo localization (MCL) are two common approaches to dealing with the global localization problem [9,10,11]. The crucial disadvantage of these two approaches is that they bear a heavy online computational burden [12], and the bigger the working space is, the more serious the problem. To solve this problem, Dellaert and Fox [13] introduced the adaptive Monte Carlo localization (AMCL) algorithm. Kullback–Leibler divergence (KLD) sampling makes the localization more efficient, so AMCL is the most widely used approach in robot localization [14]. However, if the global pose happens to be incorrect, AMCL is unable to recover from this global localization failure. Moreover, optimal filtering for non-parametric observation models that optimize the particle distribution was proposed [15], and the efficiency of global positioning was improved, but the failure rate of global localization was still very high. To enhance the performance of global positioning, Thrun [16] proposed the mixture MCL algorithm, but the two steps of sampling and calculating the importance weight increase the computational difficulty, and are very difficult to realize. Therefore, the improved MCL cannot absolutely meet the requirements of a robot in terms of the accuracy and real-time performance of global positioning.

For mobile robots, the upper limit has been reached by solely relying on Lidar and encoders for accurate positioning, and sensor-fusion-based methods are the main trends for indoor localization [17]. Multi-observation sensor resetting (MOSR) localization converges more quickly and accurately by relaxing the assumption that observations from separate frames of the camera are independent [18], but the camera is easily influenced by illumination conditions, and the positioning accuracy of robot drops when the illumination changes drastically [19]. To overcome the positioning error caused by the change of light, Choi [20] introduced an RFID system based on a tag floor using passive RFID tags for mobile robot localization. However, the average error is seriously dependent on the deployment of RFID tags because of its high revamping requirements for the environment where the service robot works. Therefore, this method is not suitable for application in service robots. Depth camera-based indoor mobile robot localization was introduced by Biswas [21], and 3D depth data were used, making the calculation load large. Although the positioning accuracy was less than 0.1 m, the global positioning efficiency was low because the positioning time reached 20 s. Luca [22] proposed a method of combining a fingerprinting algorithm based on K nearest neighbors (KNN) and a particle filter. However, the average error of this method was 1.28 m, making it unable to adapt to the precise positioning of the robot. Particle filter robot localization through robust fusion was proposed [23], but the installation of external equipment is complicated and is not conducive to the popularization of robots. Biswas [24] combined a Wi-Fi signal and Lidar data to realize the localization of a mobile robot by the particle filter algorithm, but the average convergence time was as high as 11 s, and the average positioning error reached 0.7 m.

In this paper, a new indoor localization system is proposed that fuses Wi-Fi, magnetic compass, encoders, and Lidar data and uses a particle filter and a Wi-Fi fingerprint localization algorithm, which effectively solves the problems of accuracy and efficiency in robot global positioning. The Wi-Fi fingerprint localization algorithm adopts the K nearest neighbors based on possibility (KNNBP) algorithm first created here. The probability-based similarity is used instead of the traditional Euclidean distance, eliminating the negative effects of invalid RSSI. So, it is suitable for diverse Wi-Fi environments. The Wi-Fi positioning and the compass provide more effective constraints for the particle distribution, making the robot global positioning less iterative with less resource consumption. When global positioning for the robot is required, the KNNBP Wi-Fi fingerprint localization algorithm first uses the Wi-Fi RSSI to get the global rough position of the robot, and the rough position, Lidar, compass, and grid map are used to get the precise localization of the robot according to the hybrid particle filter localization (HPFL) algorithm. In this process, the approach of uniformly distributing particles in the whole map is abandoned. Instead, only a small number of particles is maintained, which effectively improves the convergence efficiency of the particle filtering. In addition, Gaussian noise is added to the particles after converging to improve the diversity of particles, so the robustness of pose tracking is enhanced. Through the method proposed in this paper, not only the global positioning accuracy and efficiency, but also the local pose tracking of the robot is greatly improved.

The key contributions of the introduced solution can be summarized as follows:

1. An indoor localization system in a fusion of multiple sensors is designed, improving the accuracy rate and efficiency of global positioning for the indoor service robot.
2. A novel Wi-Fi fingerprint localization algorithm called KNNBP is created. Compared with the traditional fingerprinting algorithm, this algorithm adopts the similarity degree based on conditional probability instead of Euclidean distance, thus improving positioning accuracy and adaptability to diverse indoor environments.
3. HPFL is proposed, and saves computing resources compared with the traditional Monte Carlo localization. Thus, the real-time performance and accuracy are both achieved. Moreover, due to the amelioration of particle diversity, the robustness of local pose estimation is also improved.

The rest of the paper is organized as follows: In Section 2, a system overview is presented, and the methodologies proposed in this paper are presented. In Section 3, experiments are carried out in a long corridor to prove the accuracy and efficiency of the proposed system. In Section 4, discussions about the performance of the system are provided and future work is outlined. Finally, some conclusions are given in Section 5.

## 2. System Overview and Methodology

In this section, an overview of the system is presented. Then, key modules and important algorithms are described, which include Wi-Fi information preprocessing to build a fingerprint database, rough localization achieved by KNNBP, and precise localization based on HPFL.

### 2.1. System Overview

**Figure 1:**
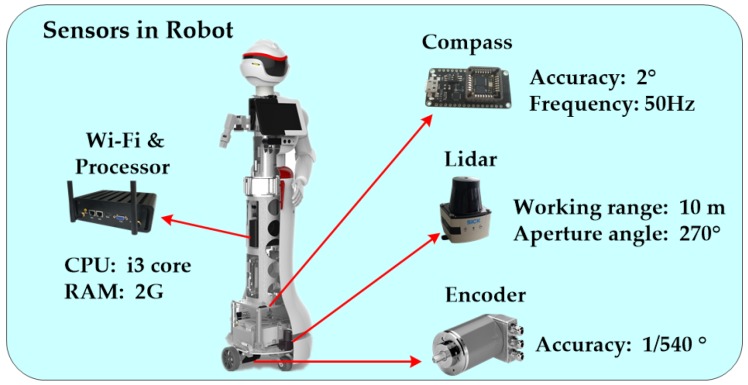
Sensors in the robot.

The multi-sensor localization system is composed of a Wi-Fi module, a Lidar unit, a magnetic compass, and encoders, as illustrated in Figure 1. We utilized a mini computer with an Intel Core i3 CPU at 2.0 GHz, 2 GB of RAM, and a solid state drive of 64 GB for processing the robot positioning algorithm, including rough localization based on KNNBP and precise localization according to HPFL. Additionally, sensor data for positioning and navigation were also collected through it. The Wi-Fi module embedded in the computer was used to collect the corresponding RSSI and MAC address of each Access Point (AP), and this information was used to establish a fingerprint database and to match the current fingerprint information with the fingerprint database during rough positioning. The magnetic compass was used to provide orientation information for the robot that is used in HPFL for precise localization, and the accuracy of this compass was 2°. The encoder provided mileage information to be used in the prediction process during which the sample motion model odometry is adopted [11], and it was also used in building a grid map. The Lidar was a SICK Tim561 laser detector with 10 m working range and 270° aperture angle. In the update stage of precise positioning based on HPFL, the detection information is mainly used to match the grid map corresponding to the environment to estimate the position and posture information of the robot, for obstacle detection and building the grid map.

**Figure 2:**
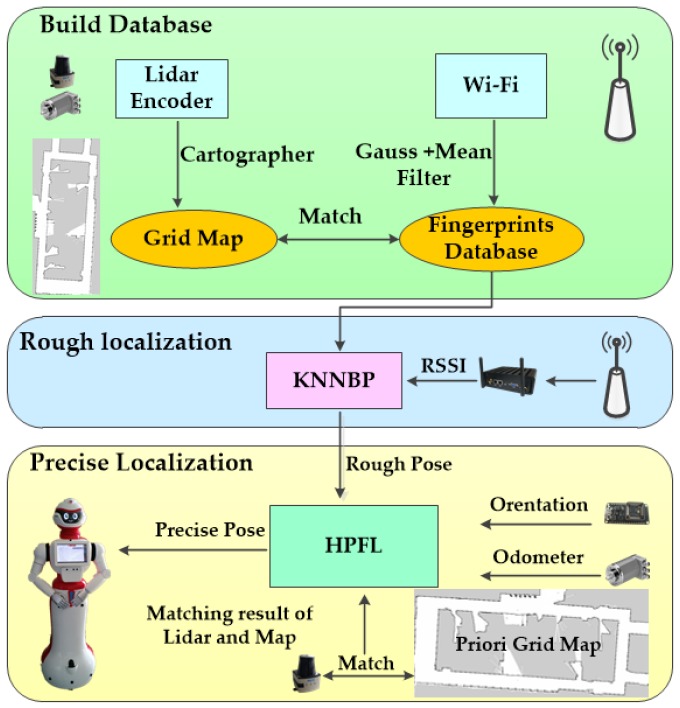
Multi-sensor fusion localization system flow. HPFL: hybrid particle filter localization; KNNBP: K nearest neighbors based on possibility; RSSI: received signal strength indicator.

The multi-sensor fusion localization system is shown in Figure 2 as the following:

Step 1: Cartographer [25] is used to establish a global 2D occupancy grid map. In the process of building a map, the center processor records a set of Wi-Fi fingerprint data every 2 m, and a Wi-Fi fingerprint database matching with the grid map is generated after processing the recorded RSSI.

Step 2: The grid map and the fingerprint database are loaded for global positioning, and the current RSSI of the robot matches with the information in the fingerprint database through the KNNBP algorithm to obtain the rough position of the robot.

Step 3: According to the rough position provided by the Wi-Fi localization and the attitude information provided by the magnetic compass, the HPFL is used to get the precise global position.

Step 4: In the process of indoor navigation, the sample motion model is used to predict the position and posture of the robot, and according to the Likelihood range finder module to update the position and posture, the accurate position of the robot is updated in real time to ensure the position tracking of the robot. To ensure the global localization, the robot is forced to repeat Step 2 every 30 min.

### 2.2. Building the Fingerprint Database

The core problem of establishing the fingerprint database is the pretreatment of the Wi-Fi signal. Although the RSSI is the most commonly used reference information, it is often prone to severe multi-path influence and measurement errors. Therefore, in practical applications, the use of preprocessing techniques on raw collected data helps the RSSI to be a suitable estimator of distance [26]. Rough localization is performed using RSSI, which is the parameter used to estimate distance between mobile and anchor nodes or APs [27]. The position information of the robot and the MAC address and RSSI of different APs are recorded at the recording positions. The RSSI of an AP is collected once every 1 s, and 200 sets of data are obtained in a position. Median filtering and mean filtering are the two most commonly used data preprocessing methods. Therefore, the effects of the two algorithms are compared, and the mean filter is

(1) $$\mathrm{RSSI}=\frac{1}{N}\sum_{i=1}^{N}{\mathrm{RSSI}}_{i}.$$

The median filter is

(2) $$\mathrm{RSSI}=\mathrm{Median}\;\mathrm{of}\;\{{\mathrm{RSSI}}_{1},{\mathrm{RSSI}}_{2},{\mathrm{RSSI}}_{3},\ldots ,{\mathrm{RSSI}}_{N}\}.$$

**Figure 3:**
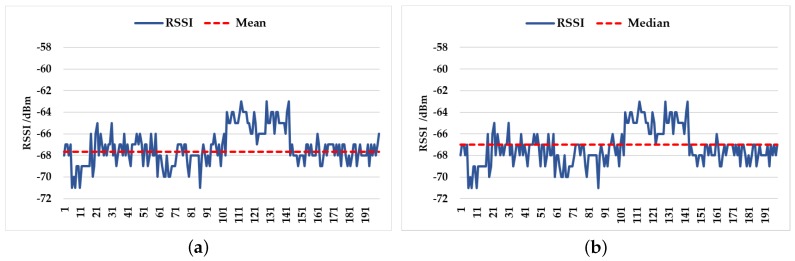
(**a**) The relationship between the RSSI set and its mean, and (**b**) the relationship between the RSSI set and its median.

As illustrated in Figure 3a, the signal always fluctuates around the mean value, so the data after the mean filtering can represent the signal intensity information of an AP. In Figure 3b, the median of the data set is significantly different from the characteristics of the data set.

**Figure 4:**
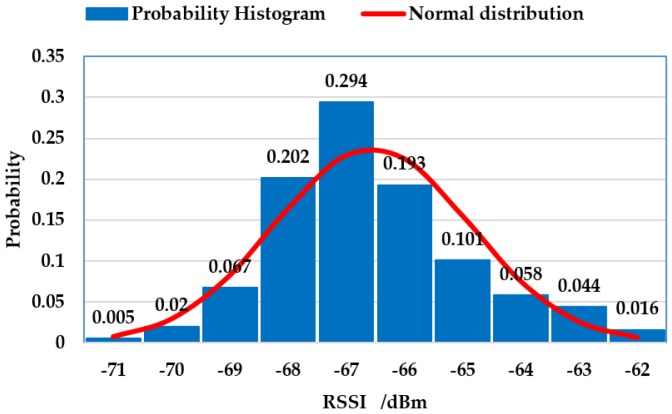
This histogram indicates the RSSI probability distribution of measured values. The *x*-axis is the intensity value of the received signal, and the *y*-axis represents the probability of the received signal intensity values in 1000 measurements. The curve is the normal distribution of the measured RSSI.

However, there are also some RSSI values that have a small occurrence probability and a great difference from the wave center of the signal intensity, because mean filtering is an extremely susceptible value of RSSI. So, using only a mean filter cannot meet the requirement of finding a value that can describe the performance of an RSSI set. Therefore, it is necessary for us to continue to do data processing for Wi-Fi fingerprint information using other methods. In Figure 4, we analyze the RSSI according to a signal intensity probability distribution histogram.

From the probability distribution histogram, we can see that the signal intensity was characterized by a normal distribution. The intensity of the signal between −68 dBm and −66 dBm appeared most frequently, and then decreased on both sides, and the frequency of signal intensity on the left and right sides of the histogram were small-probability events. To improve the reliability of the mean filtering, the intensity values of these signals were eliminated. Therefore, a Gaussian filter was introduced to eliminate the noise before performing the mean filtering [28], thus reducing the intensity error of these signals.

According to the histogram of the signal distribution, RSSI signal intensity obeys a normal distribution

(3) $$\frac{\mathrm{RSSI}-\mu }{\sigma }∼N\left(\mu ,\sigma^{2}\right),$$

where μ is the mean, and σ is the standard deviation:

(4) $$\mu =\frac{1}{n}\sum_{i=1}^{n}{\mathrm{RSSI}}_{i},$$

(5) $$\sigma =\sqrt{\left(\frac{1}{n}\sum_{i=1}^{n}{\left({\mathrm{RSSI}}_{i}-\frac{1}{n}\sum_{i=1}^{n}{\mathrm{RSSI}}_{i}\right)}^{2}\right)}.$$

To avoid over- or under-filtering, we selected the data within 90%, 80%, and 68.3% of the probability:

(6) RSSI∈(μ−1.65σ,μ+1.65σ),

(7) RSSI∈(μ−1.28σ,μ+1.28σ),

(8) RSSI∈(μ−σ,μ+σ).

**Figure 5:**
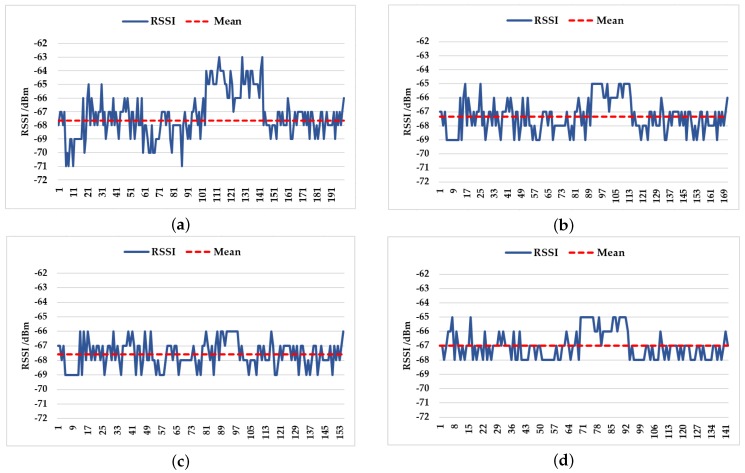
(**a**) The raw RSSI data and their mean values before Gaussian filtering. (**b**) The relationship between RSSI data within 90% of the probability and their mean. (**c**) The RSSI data within 80% of the probability and their data. (**d**) The relationship between the RSSI data within 68.3% of the probability and their mean.

In the present study, the 200 sets of data collected were processed by a Gaussian filter. According to the collected data, the mean μ was −67.25 dBm, and the variance σ was 1.66, so the final ranges of RSSI were (−70.0 dBm, −64.5 dBm), (−69.3 dBm, −65.1 dBm), and (−68.9 dBm, −65.6 dBm) when the data were within 90%, 80%, and 68.3% of the probability. By means of the mean filter after the Gaussian filter, we obtained the signal intensity change line graph as illustrated in Figure 5. It can be seen from Figure 5c that the mean of RSSI data within 80% probability could mostly depict the characteristic of the set, and after the mixed filtering method of Gaussian and mean filtering, the signal intensity fluctuated less, and the mean value could represent the whole performance of all tested RSSI values at the same position. The mean of the processed data set by Gaussian filter within 80% probability approximated the characteristics of the set. Therefore, the data set was preprocessed by a Gaussian filter within 80% probability and mean filter.

**Figure 6:**
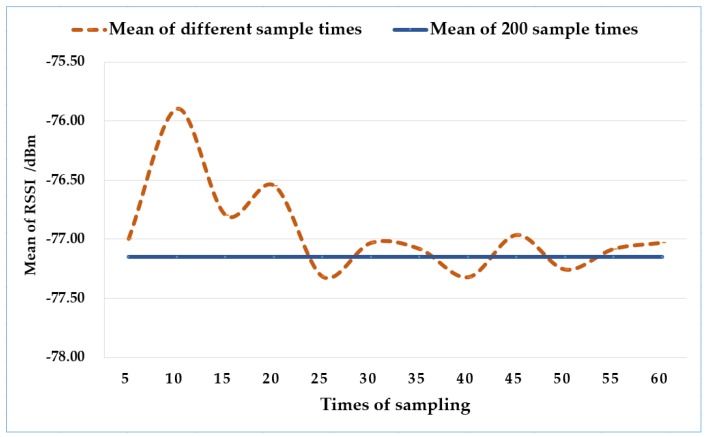
Verification of optimum sampling times.

In practical applications, 200 sampling times per location leads to an inefficient process in establishing a fingerprint database. Therefore, this paper verifies the optimal sampling times. First, 200 RSSI data were collected and processed as mentioned above at a certain position in the laboratory, and the obtained mean was regarded as a reference. Then, at the same location, the RSSIs were collected for different times and processed with the same methods as before to get the final mean, as illustrated in Figure 6. The figure illustrates that the mean value basically fluctuated near the reference value when the sampling times was greater than or equal to 30. Therefore, the optimal number of samplings was 30.

Finally, the fingerprint database has to be built. When the robot uses cartographer to construct the occupancy grid map, it optimizes the pose based on the branch-and-bound approach for computing scan-to-submap matches [25]. The robot performs a set of Wi-Fi information collection every 2 m according to the optimized position, and RSSI values are recorded every 1 s for 30 consecutive times at each recording location. Then, Gaussian and mean filtering are performed on 30 RSSI values corresponding to each AP, and the mean value is used as Wi-Fi fingerprint information for that location, such as ${\mathrm{RSSI}}_{m2}$ in Table 1, which represents the RSSI value of MAC${}_{m}$ (every AP has a MAC address) in position ($x_{2}$, $y_{2}$). A Wi-Fi fingerprint database matching with the grid map is ultimately generated. The format of the database is illustrated in Table 1, where *m* is the number of APs, *n* is the number of recorded positions, MAC is the MAC address corresponding to an AP, and (*x*, *y*) is the recorded position of the grid map matching with the environment.

### 2.3. Rough Localization

In the rough positioning stage, we denote $F_{c}$ = $\left\{\left(ma_{1},r_{1}\right),\left(ma_{2},r_{2}\right),\left(ma_{3},r_{3}\right),\ldots ,\left(ma_{m},r_{m}\right)\right\}$ as the Wi-Fi fingerprint information obtained from location points currently, where *ma* represents the MAC address of the detected AP, and *r* represents the RSSI value of the AP. Denote *FD* = $\left\{F_{1},F_{2},F_{3},\ldots ,F_{n}\right\}$ as the fingerprint database. $F_{j}$ = $\left\{\left(MA_{j1},R_{j1}\right),\left(MA_{j2},R_{j2}\right),\left(MA_{j3},R_{j3}\right),\ldots ,\left(MA_{jm},R_{jm}\right)\right\}$ ($F_{j}\in FD$) represents the fingerprint information corresponding to different location points in the fingerprint database, in which *MA* represents the MAC address of the recorded AP in *FD*, and *R* represents the RSSI value of the AP.

At the establishment stage of the fingerprint database, *n* positions are sampled, and Wi-Fi fingerprints are generated. The Wi-Fi fingerprint database is a sequence of $F_{j}$ (*j* $\in \left\{1,2,\ldots ,n\right\}$). At the online positioning stage, the robot first collects the Wi-Fi fingerprint information $F_{c}$ of the current position, then the information is matched with the fingerprint database *FD* to find the position of the pending point $F_{j}$ that is mostly similar to the Wi-Fi fingerprint information of the current position.

K-nearest neighbors (KNN) is the most basic and popular discriminative technique matching algorithm for Wi-Fi fingerprint location [29,30]. Based on a similarity measurement such as a distance function, the KNN algorithm determines the *K* closest matches in the signal space to the target. Then, the location of the target can be estimated as the average of the coordinates of the *K* neighbors. The KNN algorithm uses the Euclidean distance as the similarity as expressed by Equation (9) [31]. That is, it selects the corresponding position of the minimum *D* as the coordinates to be obtained:

(9) $$D=\sqrt{\sum_{i=1}^{m}{\left(r_{i}-R_{i}\right)}^{2}}.$$

**Figure 7:**
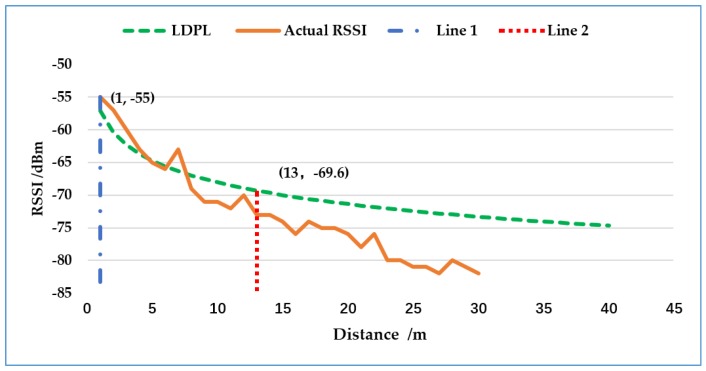
The relationship between distance and RSSI of theoretical simulation and actual measurements. LDPL: log-distance path loss.

The RSSI is a function of the distance between the transmitter and the receiving device, which varies due to kinds of in-path interference [32]. According to [33,34], the log-distance path loss (LDPL) model is typically adopted to map RSSI to propagation distance *d*:

(10) $$\mathrm{RSSI}=P_{t}-\left(PL\left(d_{0}\right)+10\cdot \xi \cdot \mathrm{lg}\left(\frac{d}{d_{0}}\right)+X_{\theta }\right),$$

where $P_{t}$ is the transmission power in dBm, *PL*($d_{0}$) is the path loss at reference distance $d_{0}$, and ξ is the path loss exponent. $X_{\theta }$ is a zero-mean normal random variable reflecting shadowing attenuation in dB. Simulation according to Equation (10) and actual measurements about the relationship between distance and RSSI are illustrated in Figure 7.

The attenuation of RSSI is affected by distance and other interference, among which the distance is the main reason when it is not too large. In the present study, since the RSSI value is regarded as the reference information of the distance, we are looking for a critical point. When the attenuation value of RSSI reaches this critical point, the attenuation of RSSI is more affected by the unexpected interference factor beyond the distance. Here, we define this attenuation value as *V* (which will be used in Algorithm 1). In Figure 7, the absolute gradient of RSSI descending between Line 1 and Line 2 is larger than that after Line 2, and both the actual RSSI and the LDPL follow the rule. So, the critical point is (13, −69.6). This means that when it is about 13 m away from the measured position (i.e., when the *V* = 15 dBm (it should have been 14.96, and approximation is taken for ease of calculation)), the influence of distance is prone to be small, and it is increased sharply by other interference. So, the RSSI value that is 13 m away from the measured position is unreasonable as the reference information of positioning.

The Wi-Fi fingerprint matching algorithm based on KNN uses all RSSI information including invalid points (e.g., the AP outside 13 m), and its RSSI value cannot be used as the information to judge the distance between the measured position and the target point, so as to introduce an error. Therefore, the accuracy and stability of the matching result are limited. In this paper, we propose a new fingerprint matching algorithm called KNNBP. Compared with the KNN algorithm, KNNBP uses a similarity degree based on conditional probability instead of Euclidean distance, which eliminates the interference of invalid APs. Finally, the normalized probability values are used as judgment information, and the reliability and accuracy are improved. The algorithm defines *p* as the similarity of each AP, and *P* represents the similarity between the current location and the location in the fingerprint database. The algorithm is presented in Algorithm 1.

**Algorithm 1** KNNBP.**Input:** *FD* = $\left\{F_{1},F_{2},F_{3},\ldots ,F_{n}\right\}$    F${}_{j}$ = $\left\{\left(MA_{j1},R_{j1}\right),\left(MA_{j2},R_{j2}\right),\ldots ,\left(MA_{jm},R_{jm}\right)\right\}$ (F${}_{j}\in$ FD)    F${}_{c}$ = $\left\{\left(ma_{1},r_{1}\right),\left(ma_{2},r_{2}\right),\ldots ,\left(ma_{m},r_{m}\right)\right\}$1:**for***j* = 1 **to**
*n*
**do**2: **for**
*i* = 1 **to**
*m*
**do**3:  **if**
*ma*${}_{i}$ = *MA*${}_{ji}$
**then**4:   **if** |$r_{i}$ − $R_{ji}$| < *V*
**then**5:     $P_{i}$ = 1 − $\frac{|r_{i}-R_{ji}|}{V}$6:   **end if**7:  **end if**8: **end for**9:   $P_{j}$ = $\frac{1}{a}\sum_{i=1}^{a}p_{i}$10:
**end for**
11: sort $P_{j}$ from largest to smallest12: select the first *K* positions ($x_{k}$,$y_{k}$) corresponding to $P_{j}$13: calculate position (*x*, *y*) = $\frac{1}{K}\sum_{j=1}^{K}\left(x_{j},y_{j}\right)$14: return (*x*, *y*)

The process of online localization defines the Wi-Fi fingerprint information at the current location as $F_{c}$, which sequentially matches with $F_{j}$ in the fingerprint database *FD*. The maximum difference is *V* = 15 dBm. As the algorithm indicates, when the MAC addresses of $F_{c}$ and $F_{j}$ are the same, if the RSSI value corresponding to the MAC address satisfies |$r_{i}-R_{ji}$| ≥ *V*, then the RSSI value is filtered out; and if |$r_{i}-R_{ji}$| < *V*, the similarity degree $p_{i}$ of the AP is calculated as

(11) $$p_{i}=1-\frac{|r_{i}-R_{ji}|}{V}.$$

The normalized similarity of the measured points in this position is as follows:

(12) $$P_{j}=\frac{1}{a}\sum_{i=1}^{a}p_{i},$$

where *a* (*a* ≤ *m*) is the number of the same MAC addresses for $F_{c}$ and $F_{j}$. The largest *K* (*K* ≥ 2) fingerprint data of *P* are obtained, and the ultimate position is the mean position of the *K* corresponding reference points, as expressed in Formula (13), where (*x*, *y*) represents the estimated position, ($x_{j}$, $y_{j}$) is the position corresponding to $P_{j}$ (*j* = 1, 2, …, *K*):

(13) $$\left(x,y\right)=\frac{1}{k}\sum_{j=1}^{K}\left(x_{j},y_{j}\right)\left(j\in K\right).$$

### 2.4. Precise Localization Based on HPFL

The average error of the Wi-Fi fingerprint localization algorithm based on KNNBP was 2.6 m. So, single Wi-Fi fingerprint localization cannot meet the navigation requirements for a robot. On the basis of the Wi-Fi fingerprint localization, the present study used the grid map, odometer (encoder data), compass information, and Lidar information to locate the robot accurately. In nonlinear dynamic models, particle filtering methods are commonly applied to develop target tracking algorithms [35,36], so the particle filter is used in HPFL to perform global positioning and pose tracking. The algorithm is presented as Algorithm 2.

The algorithm defines $\chi_{t}$ as the particle set at time *t*, *M* as the number of current particle set, $M_{x}$ is the maximum number of particles determined by the KLD sampling algorithm, and *m* represents the grid map by cartographer. The *global_flag* is the flag of global positioning, which is a Boolean value that is triggered when the robot starts and, like Step 4 of Section 2.1, fires every 30 min after starting work. Define x${}_{t}$ as the robot’s pose, $\mu_{t}$ is sensor observation, and $z_{t}$ is the measurement model at time *t*. wifi(x,y) represents the position output of rough localization, c(θ) is the orientation provided by magnetic compass, $\omega_{max}$ is the maximum weight of a particle in the particle set, $\omega_{threshold}$ is the weight threshold of a particle, and *m* is the list attribute of the object in the map environment. The precise positioning process is as follows:

**1. Prediction.** The robot predicts its position in the grid map. In line 1, when the maximum weight of the particle is below the threshold or the number of particles is less than the limited number $M_{x}$, the particle filter starts working. In lines 4 and 5, if *global_flag* is triggered, the rough pose state of the robot is predicted based on Wi-Fi and compass information. The compass provides attitude information c(θ), and Wi-Fi fingerprint information provides the position information wifi(x,y), because the angular accuracy of the compass is 2°, and the wifi(x,y) has noise itself compared with the precise position. Thus, Gaussian noise is added on the wifi(x,y) and c(θ) to improve the robustness of particles, because the accuracy c(θ) is higher than the wifi(x,y), so $\sigma_{1}<\sigma_{2}$.

(14) $$\begin{array}{c} x_{t}^{\left[M\right]}\left(\theta \right)=c\left(\theta \right)+N\left(0,\sigma_{1}\right)\\ x_{t}^{\left[M\right]}\left(x,y\right)=wifi\left(x,y\right)+N\left(0,\sigma_{2}\right) \end{array}$$

Then, a particle filter is created according to the Gaussian distribution near the rough position instead of the method of globally uniformly generating particles. When the global position is successfully obtained, the robot performs position tracking to maintain the pose to navigate. In line 7 of the algorithm, state prediction is performed according to the sample motion model odometry [11] in the position tracking phase, during which the odometry is obtained according to the encoder, and the current predicted pose of the robot to the particle $x_{t}$ is assigned. The classical AMCL does not perform pose tracking until the robot begins to move, which is not good for local positioning of the robot. In HPFL, Gaussian noise $N(0,\sigma_{3})$ is added even if the particles are converged, as expressed in Equation (15). This guarantees the diversity of particles, so accurate pose tracking is achieved even if the robot stops moving.

(15) $$x_{t}^{\left[M\right]}=sample\_motion\left(u_{t},x_{t-1}^{\left[i\right]}\right)+N\left(0,\sigma_{3}\right)$$

In the sequential importance re-sampling particle filter (SIR-PF), a duplicating particle is the main reason for loss of the particle diversity [37]. Compared with the algorithm program 5.6 proposed by Thrun [11], HPFL adds Gaussian noise to the predicted robot pose to enhance the local diversity of particles, making the robot relocate, even if it is not moving, when the error between maximum likelihood estimate (MLE) and posterior estimate is large. Thus, the robustness of position tracking is enhanced.

**Algorithm 2** HPFL.**Input:** particle set of last time $\chi_{t-1}$, orientation information *c*(*θ*), rough position *wifi*(*x*,*y*),    sensor observation $u_{t}$, measurement module $z_{t}$, grid map *m***Initilization:** current particle set $\chi_{t}=\phi$, number of current particles *M* = 0,       max number of particles $M_{x}$ = 0, max weight of M particles $\omega_{\max}^{\left[M\right]}$ = 0       the threshold of particle weight $\omega_{threshold}$1:
**while**
$M<M_{x}\;\left|\right|\;\omega_{\max}^{\left[M\right]}<\omega_{threshold}$
**do**
2: draw *i* with probability $\propto \omega_{t-1}^{\left[i\right]}$     # Particles are extracted from the particle set at time t− 13: **if** (global_flag) **then**          # If global localization is required4:  $x_{t}^{\left[M\right]}\left(\theta \right)=c\left(\theta \right)$ + N$\left(0,\sigma_{1}\right)$       # Predict orientation of particles according to compass5:  $x_{t}^{\left[M\right]}\left(x,y\right)=wifi\left(x,y\right)$ + N$\left(0,\sigma_{2}\right)$  # Predict position of particles according to Wi-Fi6: **else**7:  $x_{t}^{\left[M\right]}$ = sample_motion($u_{t}$,$x_{t-1}^{\left[i\right]}$) + N$(0,\sigma_{3}$)     # Predict position of particles according to encoder8: **end if**9: $\omega_{t}^{\left[M\right]}$ = measurement$\left(z_{t},x_{t}^{\left[M\right]},m\right)$  # Update weights of particles10: $\chi_{t}=\chi_{t}+\langle x_{t}^{\left[M\right]},\omega_{t}^{\left[M\right]}\rangle$      # Update particle set based on Importance Re-sampling11: calculate $M_{x}$ according to KLD_sampling12: mark the particles located in obstacles and filter them13: *M* = *M* + 114:
**end while**
15:select the *K* positions $\left(x_{i},y_{i}\right)$ in $\chi_{t}$ according to the largest *K* weight $\omega^{\left[M\right]}\left(i\right)$16:
**return**
$\left(x,y\right)=\frac{1}{K}\sum_{i=1}^{K}\left(x_{i},y_{i}\right)$


**2. Update.** In line 9, the sensor measurement $z_{t}$ is used at this stage to correct the belief distribution previously predicted $\omega (x_{t-1})$, and the weight of each particle is calculated. Finally, the posterior confidence of the robot is updated. A lkelihood range finder module [11] is adopted in this process to update the particle state based on the current Lidar data and the prior map. The principle of updating weights is expressed by Formula (16):

(16) $$\begin{array}{c} x_{t}^{\left[M\right]}∼p\left(z_{t}|x_{t},m\right),\\ \omega \left(x_{t}\right)=\int p(x_{t}^{M}|u_{t},x_{t-1},m)\omega \left(x_{t-1}\right)dx_{t-1}. \end{array}$$

**3. Sampling.** The sampling stage (line 11) still uses the classical KLD sampling to determine the size and distribution of the particle set. That is, the more dispersed the particles are, the more vacancies in grid map are filled, and the higher the $M_{x}$ is [14]. The calculation of $M_{x}$ is expressed by Formula (17):

(17) $$M_{x}=\frac{k-1}{2\varepsilon }{\left\{1-\frac{2}{9(k-1)}+\sqrt{\frac{2}{9(k-1)}}z_{1-\delta }\right\}}^{3},$$

where *k* is the number of non-empty particles, and $z_{1-\delta }$ is a constant of the 1−δ quantile on the standard positive distribution. Using KLD sampling not only ensures the accuracy of location, but also avoids wasting computational resources.

In line 12, the particles are further optimized according to the actual experimental conditions. During the experiment, we found that the robot often localizes its position in the occupied grid and has a very large weight, indicating that it retains certain reliable information. Here, we will mark similar particles, and “filter” is not a true deletion—the corresponding pose information is just saved but not used.The particle’s information will still be used in the next iteration updates.

**4. Pose estimation.** The particle with the largest weight is selected in each iteration. When the pose change of the particle with the largest weight is lower than the threshold set in this paper, and more than 10 consecutive position changes are within 0.05 m, it indicates that the particle representing the pose of the robot has converged. In this iteration, the *K* particles with the largest weight are selected, and the mean value of the poses is calculated. That is, the final estimated pose of the robot is (x,y), as in Formula (18):

(18) $$\left(x,y\right)=\frac{1}{K}\sum_{i=1}^{K}\left(x_{i},y_{i}\right).$$

## 3. Experiments and Results

In this section, experiments are presented to evaluate the performance of the hybrid indoor localization system compared with other methods. The experiments were mainly designed for positioning efficiency and accuracy, where efficiency means less consumption of time under the same conditions, and accuracy means smaller error and higher success rate. First, the Wi-Fi fingerprinting algorithm KNNBP for rough localization was tested and then applied to precise localization. The precise localization was improved on the basis of AMCL that is widely used in robot localization. So, experiments comparing HPFL and AMCL were performed. These experiments were carried out in a long corridor in our laboratory with an area of 702 m${}^{2}$. In addition, the performance of some localization methods obtained from references was compared with the methods presented in this paper.

### 3.1. Rough Localization

**Figure 8:**
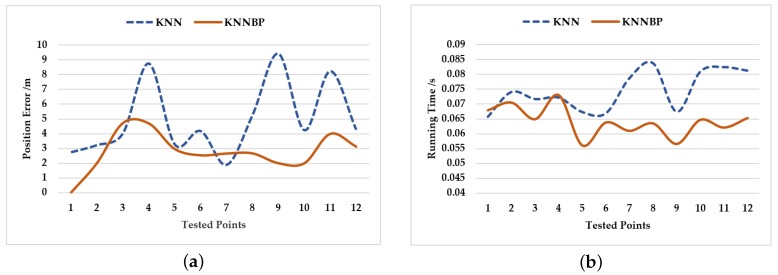
(**a**) Comparison of the positioning test between KNN and KNNBP. (**a**) Position error of rough localization according to KNN and KNNBP. (**b**) Running time for rough localization by KNN and KNNBP.

The Wi-Fi fingerprinting algorithm KNNBP was tested compared with KNN [38]. In this experiment, in order to evaluate the accuracy and efficiency of a location, 12 anchor points were selected to test the location error and time required. The time complexity of both KNNBP and KNN is O(mn), where m is the number of APs, and n is the number of recorded positions. Because KNNBP eliminates invalid data, the computation time was slightly reduced, as illustrated in Figure 8b, and the positioning accuracy was greatly improved, as illustrated in Figure 8a.

Average error (AE) and maximum error (ME) were also used to evaluate the performance of the two algorithms. The results are illustrated in Table 2. The AE and ME obtained by the KNNBP were approximately 50% of the AE and ME obtained by KNN. It can be seen that the accuracy of the robot’s rough localization was greatly improved by KNNBP.

### 3.2. Precise Localization

**Figure 9:**
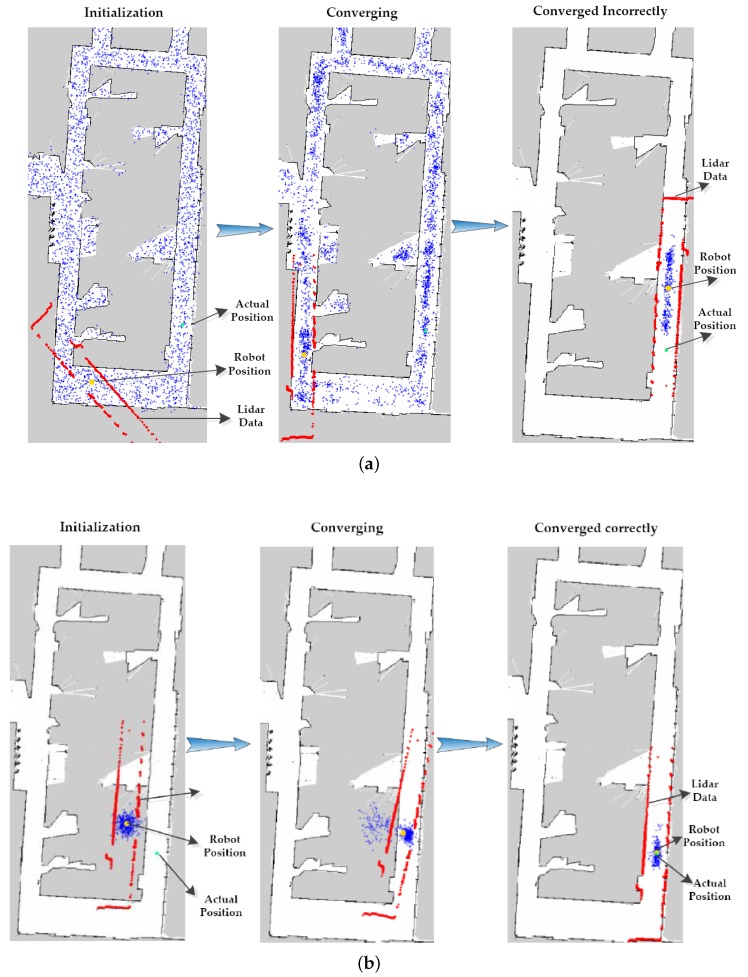
(**a**) The process of adaptive Monte Carlo localization (AMCL) with 10,000 particles was performed in a grid map with an area of 702 m^2^. Particles were initialized by a global uniform distribution method, and they then converged incorrectly. (**b**) The process of HPFL with 3000 particles in the same grid map of the actual scene. Particles finally converged successfully, and the robot got the right position.

Precise localization is mainly composed of two parts: global pose estimation and local pose tracking. The performance of HPFL was compared with the traditional localization algorithm AMCL [11]. When global positioning is triggered, the AMCL positioning adopts the global uniform particle scattering method in the global positioning as illustrated in Figure 9a, and then iteratively updates through particle filtering. The particle filter iterates continuously until the maximum weight of the particle is greater than the threshold. Finally, the particle converges to the proper location considered by the algorithm. The false convergence occurs even when the number of particles is 10,000. As is illustrated in Figure 9a, the Lidar data were not successfully matched with the map, so the pose estimation of the robot was wrong.

To prove the superiority of HPFL, a smaller number of particles was used in the experiments, as illustrated in Figure 9b. When the global positioning was performed by HPFL, the KNNBP-based Wi-Fi fingerprint location information and magnetic compass information were first integrated to provide the robot with a rough global pose, and the particles were scattered in the form of a Gaussian distribution near the pose. When the particles began to iterate, KLD sampling was used to limit the number of effective particles to improve the efficiency and ensure real-time localization. It can be seen that HPFL could ensure the accuracy of positioning even with a smaller number of particles. Finally, the Lidar data and map matched perfectly, so the robot acquired the correct pose estimation.

**Figure 10:**
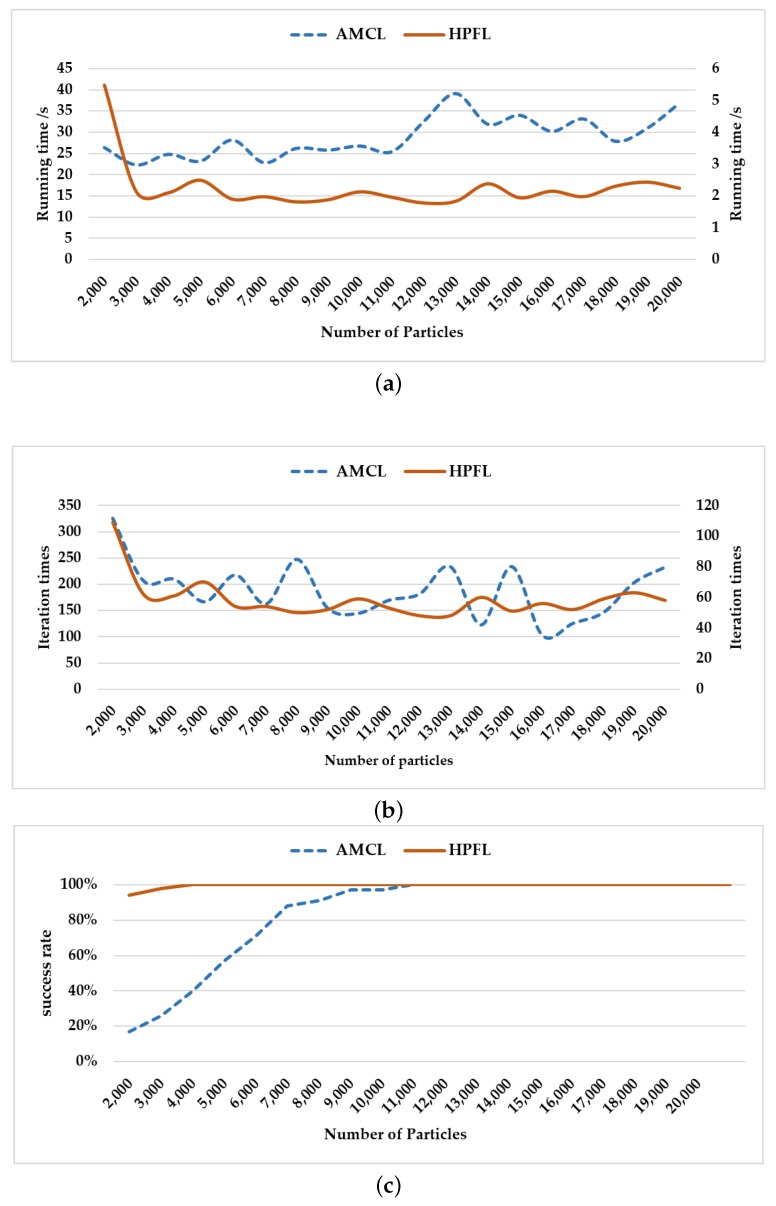
(**a**) Running time for AMCL and HPFL with different numbers of particles. The left *y*-axis is the running time for AMCL, and the right *y*-axis is the running time for HPFL. (**b**) Iteration times of AMCL and HPFL with different numbers of particles. The left *y*-axis is the number of iterations of AMCL, and the right *y*-axis is the number of iterations of HPFL. (**c**) Success rate of AMCL and HPFL with different numbers of particles.

Furthermore, experiments were designed to verify the efficiency of AMCL and HPFL with different numbers of particles. From Figure 10a,b for AMCL, it can be seen that the more particles that were adopted, the greater the number of iterations and time consumed, but the success rate was too low when the number of particles was less than 6000, as illustrated in Figure 10c, and even though the number of particles was up to 10,000, incorrect localization still occurred at low probability. When HPFL was used, the performance was better than the AMCL under the same conditions. Additionally, the success rate of localization by HPFL was 96%, even when the number of particles was 3000.

**Figure 11:**
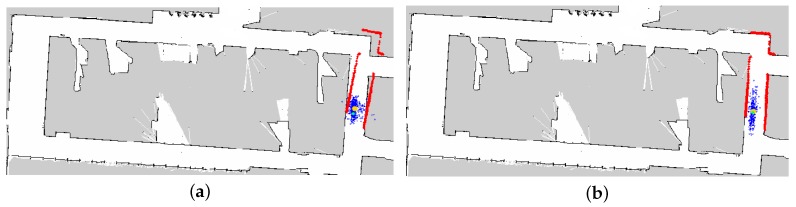
Self-correction of local pose tracking. (**a**) The position deviated when the robot rotated. (**b**) The robot corrected the pose automatically with HPFL.

In addition, HPFL prevented the particles from stopping iterations when false convergence took place, which strengthened the robustness of the local position and enabled the robot to self-correct. During the experiment, due to the low accuracy of the encoder data, the positional deviation occurred after the robot reached the target. As illustrated in Figure 11a, the robot using the AMCL could not perform self-correction if it did not move, whereas the robot using HPFL could correct itself without moving. The calibration result is illustrated in Figure 11b.

Finally, the hybrid indoor localization system that adopts KNNBP and HPFL was compared with other indoor positioning methods in terms of positioning time and error. The results indicated that the comprehensive performance in global positioning efficiency and accuracy of the method presented in this study was better than those of other methods, as presented in Table 3.

## 4. Discussion

In this paper, we presented a hybrid indoor localization system that fuses multiple sensors in order for a robot to achieve autonomous navigation. We first used a KNNBP-based Wi-Fi fingerprinting algorithm to get a rough position, and then developed an HPFL algorithm to perform precise global positioning and local pose tracking for the robot. Experiments in a real-world situation indicated that the positioning error of our solution was 0.05 m at 96% with 3000 particles, the time required for global localization was down to 1.9 s, and the robustness of local pose tracking was improved. Therefore, the hybrid indoor location system in the present study performed better than the most widely used localization algorithm.

In the future, RGBD sensors will be added to the multi-sensor localization system to achieve semantic localization. If the robot is lost or kidnapped, Wi-Fi is not the only reference to provide rough position. Trained landmarks through deep learning by RGB images and point clouds can also help the robot to globally localize accurately, so that the robot can ensure the accuracy and stability of the positioning in highly crowded places.

## 5. Conclusions

The accuracy and efficiency of localization are very important for indoor robots, and our method helps to solve these problems. The hybrid indoor localization system proposed in this paper combines Wi-Fi fingerprinting with a particle filter to achieve remarkable results. To the best of our knowledge, the KNNBP-based Wi-Fi fingerprinting algorithm was first created in the present study, and the HPFL-based particle filter is an improvement on the basis of AMCL. Our localization system that fuses various sensors not only improves the global positioning but also improves the robustness of local position tracking. The experiments indicated that our positioning method was superior to the traditional location systems in both accuracy and efficiency.

## Figures and Tables

**Table 1:** Format of the Wi-Fi fingerprint database.

	($x_{1}$, $y_{1}$)	($x_{2}$, $y_{2}$)	($x_{3}$, $y_{3}$)	…	($x_{n}$, $y_{n}$)
MAC${}_{1}$	${\mathrm{RSSI}}_{11}$	${\mathrm{RSSI}}_{12}$	${\mathrm{RSSI}}_{13}$	…	${\mathrm{RSSI}}_{1n}$
MAC${}_{2}$	${\mathrm{RSSI}}_{21}$	${\mathrm{RSSI}}_{22}$	${\mathrm{RSSI}}_{23}$	…	${\mathrm{RSSI}}_{2n}$
…	…	…	…	…	…
MAC${}_{m}$	${\mathrm{RSSI}}_{m1}$	${\mathrm{RSSI}}_{m2}$	${\mathrm{RSSI}}_{m3}$	…	${\mathrm{RSSI}}_{mn}$

**Table 2:** Comparison of error evaluation. AE: average error; ME: maximum error.

Evaluation (m)	KNN	KNNBP
AE	4.95	2.6
ME	9.43	4.73

**Table 3:** Performance of indoor localization methods.

Localization Methods	Time (s)	Error (m)	Processor
AMCL	25.6	0.05	Core i3
MOSR [18]	31	0.22	Core i5
KNNBP+HPFL	1.9	0.05	Core i3
WVT-bootstrap [33]	0.12	2	Core i5
Method in [24]	11	0.7	A laptop
FSPF [21]	20	0.1	Not public
